# Cure of Refractory Hypotension in a Hemodialysis Patient

**DOI:** 10.7759/cureus.41942

**Published:** 2023-07-16

**Authors:** Prajjwol D Bhatta, Stephen Silver

**Affiliations:** 1 Internal Medicine, Rochester Regional Health, Rochester, USA; 2 Nephrology, Rochester Regional Health, Rochester, USA

**Keywords:** arteriovenous fistula, dialysis, fistula blood flow, hypotension, high output cardiac failure

## Abstract

We present a case of a 67-year-old male with end-stage renal disease (ESRD) on hemodialysis who was admitted to the hospital after recurrent falls secondary to postural hypotension. He was not able to tolerate fluid removal on dialysis due to persistent hypotension despite maximal doses of midodrine and developed severe edema. A right heart catheterization revealed raised biventricular filling pressure consistent with right heart failure with low systemic vascular resistance and pulmonary hypertension. Duplex ultrasound of the left arm cephalic arteriovenous fistula (AVF) revealed a blood flow of 5.6 L/min. We hypothesized the cause of his high output heart failure from an AV fistula despite the lack of an increase in blood pressure after compression of the AVF. The AVF was ligated and a tunneled hemodialysis catheter was placed. Immediately after ligation, the patient was able to tolerate fluid removal with dialysis without hypotension, leading to a significant improvement in his edema and shortness of breath. This case highlights the potential adverse cardiovascular effects of AVF and the salutary effects on ligation in appropriate clinical settings.

## Introduction

An arteriovenous fistula (AVF) is the preferred method for long-term hemodialysis in end-stage renal disease. The ability to maintain long-term patency, deliver a high flow rate for effective hemodialysis, and suitability for repeated cannulation are the characteristics of an ideal fistula [1]. However, a fistula carries risks of complications like aneurysm formation, thrombosis, infection, ischemic steal syndrome, venous hypertension, and neuropathy [2,3]. More recently, adverse cardiovascular consequences of AVFs have been recognized. These include pulmonary hypertension, and high-output cardiac failure (HOHF) [4].

Here, a patient is described with refractory hypotension and volume overload that was addressed successfully by ligation of his AVF.

## Case presentation

**Figure 1:**
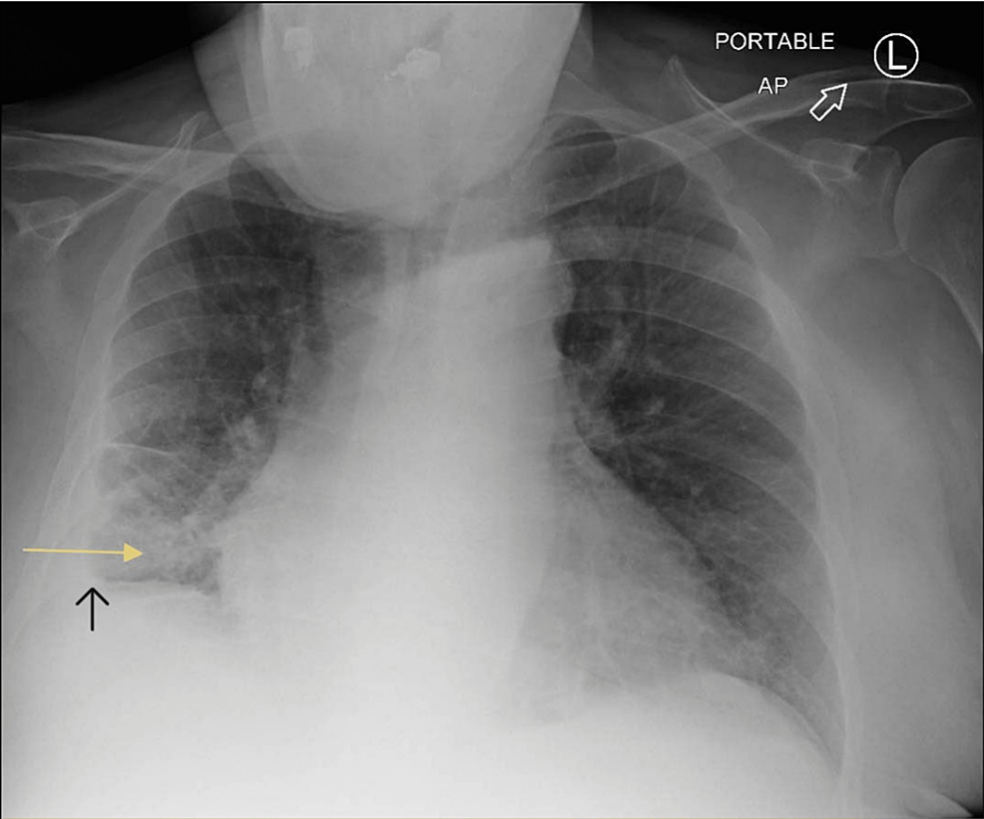
Chest X-ray The yellow arrow shows right base airspace disease and the black arrow shows right-side pleural effusion

The patient is a 67-year-old male with a past medical history of heart failure with preserved ejection fraction (HFpEF), end-stage renal disease (ESRD) secondary to diabetic nephropathy, peripheral vascular disease (PVD), atrial fibrillation (AF), type 2 diabetes mellitus (DM), obstructive sleep apnea (OSA), and aortic stenosis status post-transcatheter aortic valve replacement (TAVR). He had a left arm cephalic AVF placed four years ago. He presented to the hospital after falls and passing out due to postural hypotension. On examination, heart rate was 110/min and irregular, blood pressure 94/60 (millimeter of mercury) mmHg, oxygen saturation 97% on 2 L/min oxygen, and respiratory rate of 18 breaths per min. Jugular venous distension was present and there was severe bilateral lower and upper limb edema. There were bilateral crackles on the auscultation of the lungs. The heart sounds were normal. The AVF had a continuous bruit and thrill. Hemoglobin ranged from 8.8 to 9.2gm/dl. Electrolytes were unremarkable. Chest X-ray showed right-side pleural effusion with right base airspace disease (Figure 1).

During his hospital course, amiodarone was initiated for the control of AF. Midodrine was progressively increased to 30 mg three times a day, and metoprolol was reduced. Despite these interventions, intradialytic systolic blood pressure dropped to the range of 70 mmHg-80 mmHg limiting fluid removal often to 1 L or less. He had symptoms of dyspnea and orthopnea requiring supplemental oxygen. His quality of life was poor and the palliative care team was involved in goals of care discussions.

**Figure 2:**
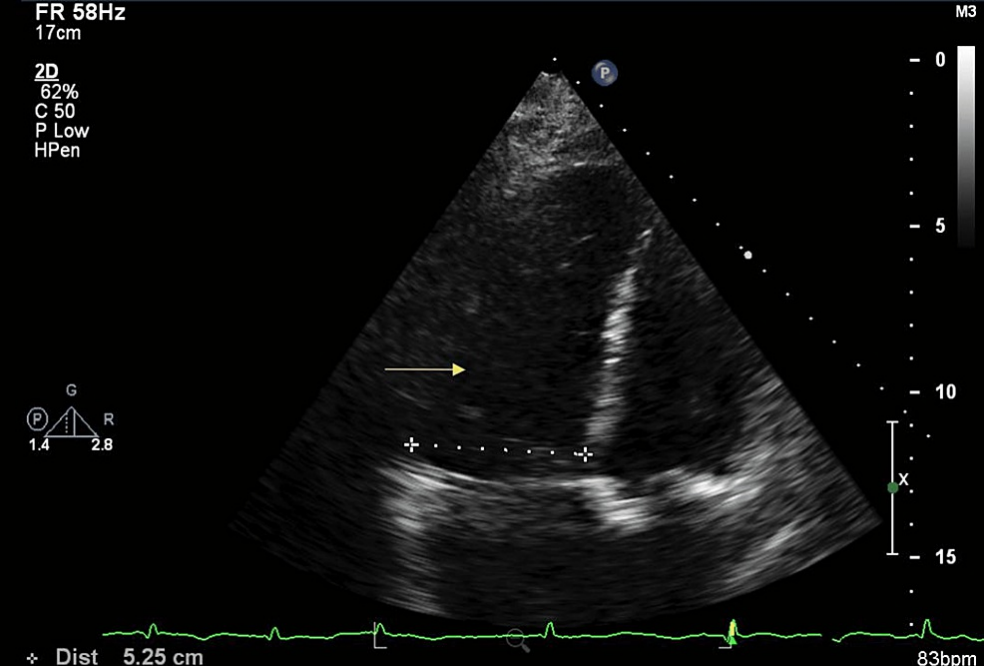
Echocardiography The yellow arrow shows the severely dilated right ventricle

**Figure 3:**
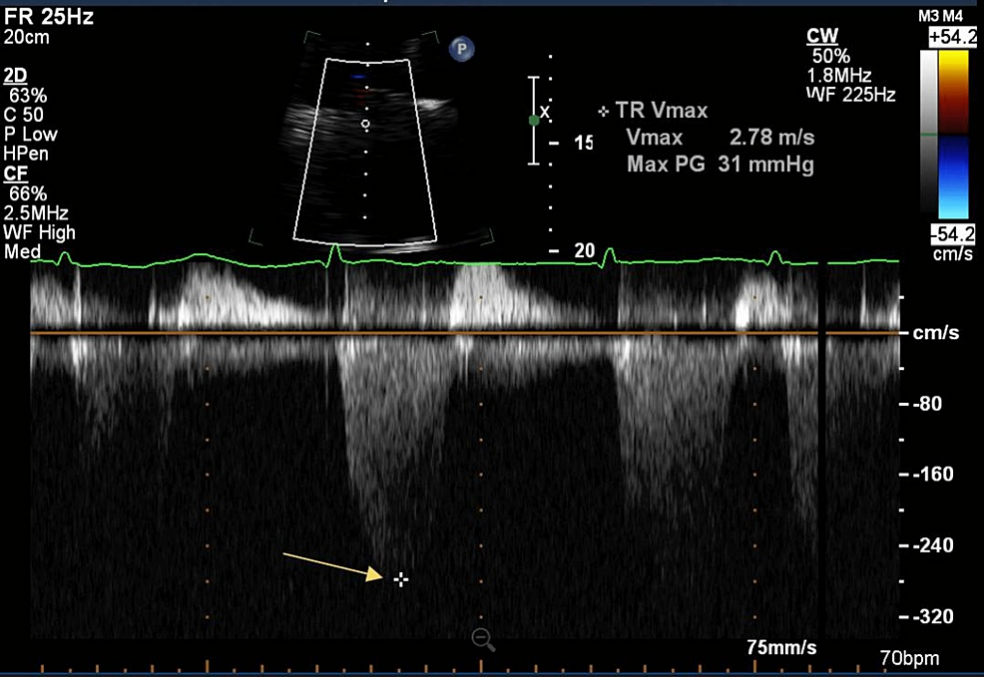
Echocardiography The yellow arrow shows a tricuspid regurgitation velocity maximum of 2.78 m/s

He underwent echocardiography, which disclosed normal left ventricle (LV) size with preserved systolic function with ejection fraction (EF) of 65-70%, severely dilated right ventricle (RV) with severely reduced systolic function (Figure 2), an estimated RV systolic pressure of 49 mmHg suggestive of mild pulmonary hypertension (Figure 3). Right heart catheterization showed right atrial pressure of 25 mmHg, RV pressure 85/25 mmHg, pulmonary artery (PA) pressure: 85/40/56 mmHg, systemic vascular resistance (SVR) 720 dyn·s·cm-5, pulmonary capillary wedge pressure (PCWP) of 27 mmHg, Fick cardiac output (CO) 7.1 L/min, and cardiac index (CI) of 2.5 L/min/m^2^. In sum, there was evidence of severe elevated right and left-sided filling pressure, low SVR, severe pulmonary hypertension, and normal CI.

**Figure 4:**
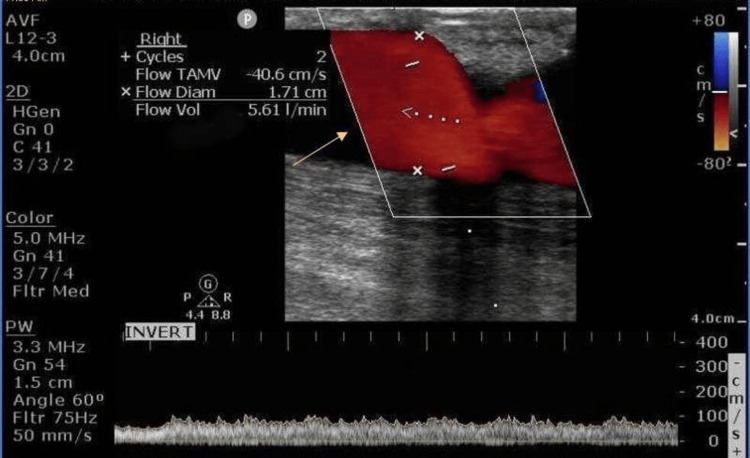
Duplex ultrasound The yellow arrow shows the blood flow in the left arm fistula

Duplex ultrasound of the fistula revealed blood flow of up to 5.6 L/min, which was significantly elevated (Figure 4). Evaluation for the Nicoladoni-Branham sign [5], in which systolic blood pressure after AV fistula occlusion with blood pressure cuff for 30 seconds was performed. It refers to the bradycardia and hypertension noted when the artery proximal to an AV fistula is compressed. Systolic blood pressure after measures of three blood pressures in a row averaged systolic 95 mmHg without occlusion, and the average systolic blood pressure of three blood pressure readings after occlusion of the AV fistula was 99 mmHg, a minimal increase.

Despite this, it was hypothesized that the fistula was a major cause of the patient’s low SVR and high cardiac demand. The decision was made to ligate the AV fistula in the hope of correcting refractory hypotension and heart failure. Vascular surgery ligated the AV fistula after the placement of a tunneled dialysis catheter.

On the day of fistula ligation, 2 L of fluid was removed with dialysis, without hypotensive episodes. Subsequent efforts of volume removal with dialysis were successful. The patient’s weight decreased by 12 kg on the tenth day after fistula ligation accompanied by a decrease in edema, dyspnea, oxygen requirements, and the need for blood pressure support with midodrine.

## Discussion

We present a patient with ESRD on hemodialysis with debilitating hypotension and heart failure whose clinical course was dramatically improved by ligation of his AVF. There were accurate hemodynamic measures of cardiac function in our patient prior to ligation that were consistent with the expected untoward effects of the AVF. Temporary occlusion of the AVF, which was carefully performed, did not predict the salutary response to ligation of the AVF in our patient. Thus, the utility of this measure appears limited.

An AV fistula created for hemodialysis access results in direct communication of a high-pressure, high-resistance circuit with a low-pressure low-resistance circuit, resulting in increased blood flow (Qa). As a consequence, there is an increase in wall shear stress (WSS) on endothelial cells that increase chemical mediators like nitric oxide (NO), an endothelium-derived hyperpolarizing factor, leading to dilation of the fistula and normalization of WSS [6]. This adaptive remodeling occurs to restore vessel shear and tensile stresses back to baseline.

The incidence of high-output failure due to AVF has not been well-defined [7]. The risk factors for HOHF are male sex, upper arm AVF, vascular access blood flow (Qa) >2.0 L/ min, and history of vascular access surgery [8]. HOHF is more likely to occur in patients with preexisting heart disease like congestive heart failure (CHF), left ventricular hypertrophy (LVH), coronary artery disease (CAD), or pulmonary hypertension (PHTN) [9].

It has been observed that the relation between blood flow in the fistula and CO is non-linear; there is an initial plateau of CO followed by a steep slope of CO at high blood flow in the fistula, which is beyond 2.2 L/min [10]. Our patient’s estimated AV fistula blood flow on duplex US was 5.6 L/min. The ratio of Qa/CO >0.3 is also considered a risk factor for HOHF and decompensation [11]. The ratio of Qa/CO in this patient was 0.78.

The AV fistula and peripheral circulation are arranged in a parallel circuit. SVR is reduced and, as a result, heart rate, contractility, systemic filling pressure, and CO all increase. The AVF may result in dilatation and hypertrophy of LV secondary to an increase in CO. There is evidence that LVH is strongly associated with an increase in the risk of mortality and cardiovascular events, including sudden cardiac death in ESRD patients [12]. An increase in venous return from AVF results in high blood volume in the pulmonary artery. Further, endothelial dysfunction in ESRD causes a low level of circulating nitric oxide contributing to an increase in PA pressure, as additional CO could not be accommodated, resulting in pulmonary hypertension.

Managing HOHF secondary to an AV fistula is a unique challenge. Ideally, we wish to not only preserve the AVF but also treat heart failure. Some modalities of treatment for HOHF from an AVF are ligation of the fistula, a banding technique to constrict the fistula at one or more places to decrease the blood flow, use of hemoclips downstream to the venous supply to increase the resistance, and ligating the brachial artery anastomosis and attaching a synthetic graft from the cephalic vein to the smaller diameter distal radial artery in upper arm fistulas [13,14]. In our patient, the severity of his clinical condition and high risk for surgery led our team to choose ligation.

It has been demonstrated in a randomized controlled trial that ligation of AVF decreased LV mass significantly [15]. There is also a significant decrease in right atrial and right ventricular diameter after AVF ligation [16]. Furthermore, ligation of AVF reduces pulmonary blood flow, which may also reduce pulmonary artery pressure. Hence, patients with severe pulmonary hypertension may also benefit from AVF ligation. The diagnosis of pulmonary hypertension should be considered in any patient with AVF and shortness of breath.

The burden of HOHF secondary to AVF is under-recognized. Guidelines have suggested regular monitoring of dialysis patients with AVF flow >1500 ml/min with echocardiography and clinical assessment for heart failure [17]. The potential for adverse cardiovascular consequences should also be considered before an AVF is placed. It has been proposed that in patients with advanced heart failure, AVF placement should be avoided, and in patients with less advanced heart failure, placement of a distal arm AVF is preferred over an upper arm AVF [18].

## Conclusions

Before or after an AVF is placed, the ill effects of an AVF should be considered in any hemodialysis patient with heart failure, pulmonary hypertension, or low blood pressure. The lack of hemodynamic effects after occlusion of the AVF should not rule out the potential salutary effects of revision or ligation of the AVF.
